# Hydrogen isotope analysis in W-tiles using fs-LIBS

**DOI:** 10.1038/s41598-023-29138-2

**Published:** 2023-02-09

**Authors:** Steffen Mittelmann, Kévin Touchet, Xianglei Mao, Minok Park, Sebastijan Brezinsek, Georg Pretzler, Vassilia Zorba

**Affiliations:** 1 Institute of Laser and Plasma Physics, Heinrich-Heine University Düsseldorf, 40225 Düsseldorf, Germany; 2 Laser Technologies Group, Lawrence Berkeley National Laboratory, Berkeley, CA 94720 USA; 3 Forschungszentrum Jülich GmbH IEK-4 Plasmaphysik, 52425 Jülich, Germany; 4 Department of Mechanical Engineering, University of California at Berkeley, Berkeley, CA 94720-1740 USA

**Keywords:** Energy science and technology, Materials science, Physics

## Abstract

Laser-Induced Breakdown Spectroscopy (LIBS) is a promising technology for in-situ analysis of Plasma-Facing Components in magnetic confinement fusion facilities. It is of major interest to monitor the hydrogen isotope retention i.e. tritium and deuterium over many operation hours to guarantee safety and availability of the future reactor. In our studies we use ultraviolet femtosecond laser pulses to analyze tungsten (W) tiles that were exposed to a deuterium plasma in the linear plasma device PSI-2, which mimics conditions at the first wall. A high-resolution spectrometer is used to detect the Balmer-$\alpha$ transition of the surface from implanted hydrogen isotopes (H and D). We use Calibration Free CF-LIBS to quantify the amount of deuterium stored in W. This proof-of-principle study shows the applicability of femtosecond lasers for the detection of low deuterium concentration as present in first wall material of prevailing fusion experiments.

## Introduction

The Plasma-Facing Components (PFCs) of a magnetically confined fusion vacuum chamber are exposed to extreme environmental conditions including extraordinary high temperature, radiation, and high energy particle fluxes. All those circumstances will lead to surface erosion, particle deposition, and potentially destruction with a resulting higher likelihood of fuel retention during the fusion-plasma operation^1–3^. To ensure the safety and tritium self-sufficiency of an upcoming fusion reactor, the total absorbed amount of deuterium and tritium inside of the PFCs need to be tracked in-situ over many operation hours. The use of Laser-Induced Breakdown Spectroscopy (LIBS) has been proposed^4^, as it has also numerous applications in hands-off, low invasive diagnostics like nuclear waste management^5^ or material analysis in current and upcoming mars missions^6^. Especially, when it comes to the detection of minor elements and high depth resolution applications, LIBS appears as a powerful tool^7–9^. One requirement for a quantitative LIBS method is a reduced heat diffusion to the bulk material by the laser pulses so that stoichometric approximations might hold, when the expanding plasma is analyzed. To ensure this and to achieve a high depth resolution, the use of a laser pulse duration shorter than picoseconds is a preferred solution^10^.

In this work we used UV ultra-short laser pulses for laser plasma generation in an argon environment, coupled with optical emission detection with a high spectral resolution Czerny-Turner spectrometer. The detection method here is similar to studies by Kurniawan et al.^11^. This system capabilities combined with a CF-LIBS approach allowed detection and quantification of the hydrogen and deuterium content of tungsten tiles exposed to a deuterium plasma in the linear plasma device PSI-2 at Forschungszentrum Jülich^12^. These tiles serve as surrogates for PFCs in this context. The deuterium content calculated with CF-LIBS was directly compared to results obtained with Thermal Desorption Spectroscopy (TDS). The UV wavelength of $343\,{\mathrm{nm}}$ and the pulse duration of $500\,\mathrm{fs}$ were chosen to work towards the highest possible depth resolution, which is promising due to the small optical penetration depth of $7.4\,{\mathrm{nm}}$ in tungsten^13^. This work serves as a proof-of-principle for in-situ quantification of isotopes of hydrogen for future application in plasma-facing components in confinement fusion experiments.

## Results and discussion

**Figure 1:**
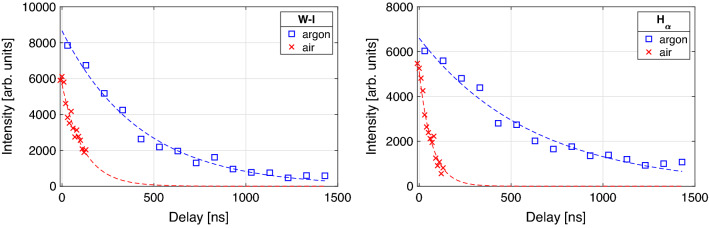
Detected maximum count (gate width $100\,{\mathrm{ns}}$) of the W-I (left) and $\text{H}_{\alpha }$ (right) spectral lines at $643.97\,{\mathrm{nm}}$ and $656.28\,{\mathrm{nm}}$ on untreated tungsten (W) tile using the same setup with ambient argon (blue squares) and air (red crosses) at atmospheric pressure. Exponential fits are indicated with dashed lines.

The following experiments are executed with the setup described in detail in the methods section. Here, an argon gas flow, according to similar findings^14^, is used to enhance the observed plasma emission. The heavier Ar atoms in the ambient gas (compared to nitrogen atoms in air) around the expanding plasma lead to a longer plasma persistence. This and a higher plasma temperature lead to stronger line emission. Figure 1 presents the temporal evolution of the emission of neutral tungsten (W-I at $643.97\,{\mathrm{nm}}$) and hydrogen Balmer-$\alpha$ ($\text{H}_\alpha$ at $656.28\,{\mathrm{nm}}$) from the tiles surface using air and an argon flow in the ambient atmosphere. We observe an increase in half-life of more than a factor of two. This results in a significant signal enhancement. The electron number density $n_e$ and heavy particle temperature $T_h$ in the plasma are determined by observing the $\text{H}_\alpha$ peak. The FWHA (Full Width at Half Area) line broadening contains information on $n_e$ using equation (5) and the Doppler width (4) is used to determine $T_h$. This temperature can be compared to the electron temperature in the plasma $T_e$ determined by the Boltzmann-plot method. In Local Thermodynamic Equilibrium (LTE) conditions these temperatures can be assumed to be equal $T_h=T_e=T$.

**Figure 2:**
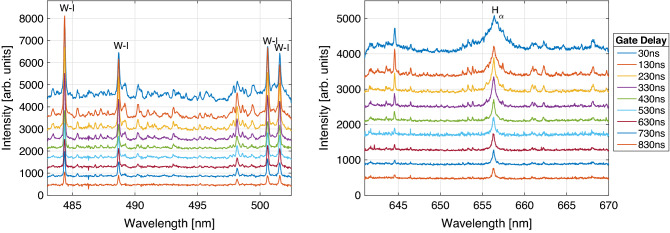
Time resolved emission from the tungsten sample under fs laser irradiation in argon. The gate width is set to $100\,{\mathrm{ns}}$ and the laser fluence is determined as $31\,{\mathrm {J/cm}}^2$. For visibility 400 counts are added across two distinct spectral ranges for each given gate delay.

Before we discuss the experimental requirements that are necessary to observe the deuterium impact on the exposed tungsten tiles, the temporal optical emission of the laser-induced plasma in this experiment is presented using a laser fluence of $31\,{\mathrm {J/cm}}^2$. This value is significantly above the ablation threshold of tungsten $F_{th}=(0.07\pm 0.06)\,\mathrm {J/cm^2}$, determined according widely applied procedure^13,15–17^. Figure 2 shows the temporal evolution of the spectra observed in this configuration with a spectrometer of instrumental broadening $w_{inst}=52\,{{\mathrm{pm}}}$ (Gaussian width) at a slit size of $100\,\mu \mathrm{m}$. Here, the instrumental broadening of the used device is determined by the spectral lines widths of a low pressure iron (Fe) Hollow Cathode Lamp (HCL). The spectral lines used for the Boltzmann-plot method are given in table 1.

**Table 1:** Spectroscopic parameters of lines selected from atomic tungsten (W-I) and the hydrogen isotope lines ($\text{H}_\alpha$, $\text{D}_\alpha$) taken from the atomic spectra database NIST^18^.

Species	$\lambda _{ki} [{\mathrm{nm}}]$	$A_{ki} [\mathrm {s^{-1}}]$	Acc. (%)	$g_k$	$E_k [{\mathrm{eV}}]$
W-I	484.381	$1.9\times 10^6$	$<10$	5	2.97
W-I	488.6899	$8.1\times 10^5$	$<10$	11	3.31
W-I	500.6150	$1.2\times 10^6$	$<10$	7	3.25
W-I	501.5304	$5.4\times 10^5$	$<10$	9	3.07
W-I	643.970	$1.29\times 10^5$	$\le 25$	9	4.71
$\text{H}_\alpha$	656.28	$4.41\times 10^7$	$\le 0.3$	18	12.09
$\text{D}_\alpha$	656.10	$4.41\times 10^7$	$\le 1$	18	12.09

Here, the transition wavelength $\lambda _{ki}$ from the upper energy level $E_k$ are given with the corresponding transition probabilities $A_{ki}$ and statistical weights $g_k$ including the corresponding accuracy.

**Figure 3:**
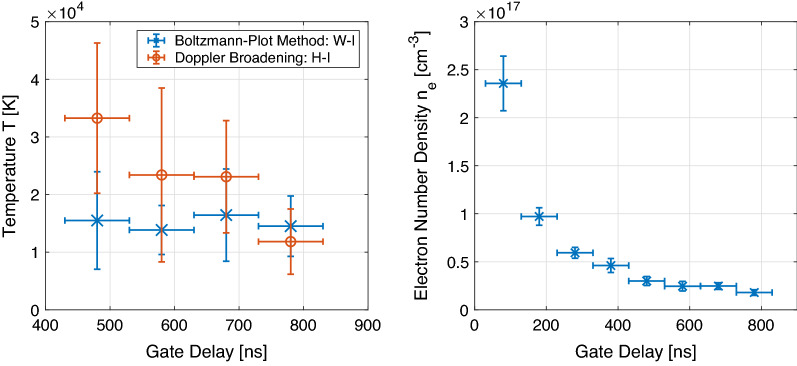
Left: Plasma temperature *T* evaluated from the observed tungsten (W) spectrum by using the Bolzmann-plot method including atomic W-I lines in blue crosses and the Doppler Broadening of the $\text{H}_{\alpha }$ line in orange circles. Right: Electron number density $n_e$ evaluated by Stark broadening of the same line. Error-bars indicate statistical deviations from the measured values on the y-axis and the used gate width on the x-axis.

Moreover, we can observe several atomic tungsten lines and the hydrogen Balmer-$\alpha$ transition ($\text{H}_\alpha$). The temperatures, calculated using the Boltzmann-plot method and the Doppler Broadening, similar to other recent evaluations of LIBS data^19–21^, are given in Fig. 3 (left) for gate delays larger than $400\,{\mathrm{ns}}$. The electron number density is evaluated by observing the FWHA of the $\text{H}_\alpha$ line and is exponentially decreasing from $2.5\times 10^{17}\,{\mathrm{cm}}^{-3}$ by one order of magnitude in the first $800\,{\mathrm{ns}}$ as shown in Fig. 3 (right). Note that the observed hydrogen Balmer-$\alpha$ line interferes with a tungsten line at $656.32\,{\mathrm{nm}}$ and the weak signal from the low concentration deuterium $\text{D}_\alpha$ at $656.1\,{\mathrm{nm}}$. Most likely, the pseudo-Voigt fit is influenced by these disturbances and the calculated temperature and density values may be overestimated.

Summing up the result of the plasma observation from the given material, the plasma temperature and number density decreases with time as expected. The parameters are in the range of $T_e\approx 15,000\,\mathrm{K}$ to $T_e\approx 10,000\,\mathrm{K}$ (Boltzmann-plot method) and $n_e\approx 2.5\times 10^{17}\,{\mathrm{cm}}^{-3}$ to $n_e\approx 10^{16}\,{\mathrm{cm}}^{-3}$. Note that the evaluated temperatures for delays lower than $400\,{\mathrm{ns}}$ are strongly deviating from reasonable values, that is why they are not plotted here. One reason for this is the high plasma density in the early expansion phase that brings a higher self-absorption and effects like opacity broadening^22^ leading to an overestimation of the spectral width of the hydrogen emission. Another explanation might be given by the differences in the first ionization potential of tungsten ($7.86\,{\mathrm{eV}}$) and hydrogen ($13.598\,{\mathrm{eV}}$) that influences the atom to ion proportion according to the Saha equation. As an example, the ionization rate $N_{ion}/N_{tot}$ is significantly higher for tungsten (factor 20) with $T\approx 12,000\,\mathrm{K}$ and $n_e\approx 10^{17}{\mathrm{cm}}^{-3}$. Similar observations were made by Giacomo et al. (2008)^23^ who observed the hydrogen emission in an aluminum plasma. The higher influence of the continuum radiation can also be observed in this regime, as well as a high plasma speed that can lead to blue shifts, slight broadening, and line asymmetry. Hence, the plasma parameters observed after $400\,{\mathrm{ns}}$ are typical values for laser-induced plasmas in an argon environment^24^. One important takeaway from the presented measurement is the transient character of the plasma observed by the exponential decrease of the number density. This is also the critical point that has to be considered, when we apply a LTE to the observed plasma. The question is whether the relaxation time $\tau _{rel}$ and corresponding diffusion length $\lambda =(D_h\cdot \tau _{rel})^{1/2}$ can be covered^25^. Here, $D_h$ is the material dependent diffusion coefficient. Typical values for metals are on the order of $\tau _{rel}\sim 10^{-9}\,\mathrm{s}$ and $\lambda \sim 10^{-5}\,\mathrm{m}$. The plasma persistence (about a few micro seconds) and plasma size (even larger than the beam diameter $\sim 20\,\mu \mathrm{m}$) of the observed expansion suggests that a LTE is reasonable in the recombination part of the process. Moreover, the McWhirter criterion in Eq. (2) is a necessary but not sufficient condition to be fulfilled. As an example it is calculated for the tungsten plasma observed with a delay of $830\,{\mathrm{ns}}$: $T_e\approx 10,000\,\mathrm{K}, n_e\approx 4.7\times 10^{16}\,{\mathrm{cm}}^{-3}$ and $\Delta E_{mn}\approx 3\,{\mathrm{eV}}$ fulfills the condition as $n_e>4.3\times 10^{15}\,{\mathrm{cm}}^{-3}$.

**Figure 4:**
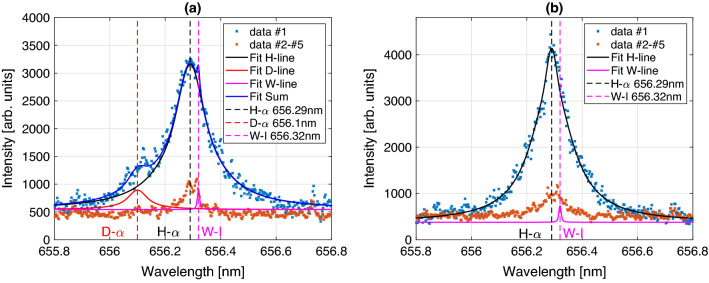
Left: High resolution detection of hydrogen isotope lines ($\text{H}_\alpha$ and $\text{D}_\alpha$) in the exposed tungsten tile. Right: Comparable record on a pure tungsten tile. In black, red, and blue pseudo-Voigt fits of the overlapping deuterium and hydrogen spectral line of Balmer-$\alpha$ transition are given. The blue crosses represent the measurement of the first laser pulse (average of 80 positions) and orange the second to fifth pulse at the same positions respectively. Colored in magenta the neutral W-I line at $656.32\,{\mathrm{nm}}$ with fixed width and amplitude is given. The applied gate delay and width are chosen as $1.08\,\mu \mathrm{s}$.

**Figure 5:**
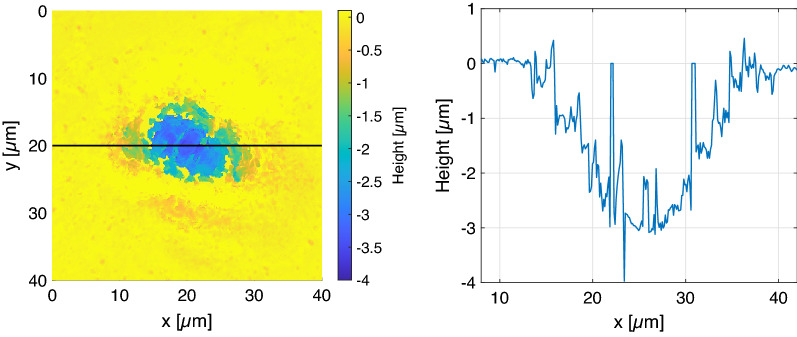
Left: Height map measurement with a white light interferometer to analyse the crater shape of five pulses on the same position on the W tile. The crater area is shown as a pseudo color surface plot. Right: Lineout in the center (along the black line) of the crater.

With this knowledge about the process, we proceeded with the detection and evaluation of the hydrogen Balmer-$\alpha$ transition from the tungsten tile with the high resolution spectrometer (instrumental broadening $w_{inst}=12.7\,{\mathrm{pm}}$ at slit width of $120\,\mu \mathrm{m}$ determined by a deuterium lamp). In Fig. 4 the detected LIBS signals of a tungsten tile exposed to the deuterium plasma in PSI-2 as in Jiang et al. (2021)^12^ is compared to an unexposed tile. In blue the accumulated LIBS emission data from 80 independent single pulse interactions on the unperturbed sample surfaces are plotted and in orange the sum of all measurements from laser pulse number 2 to 5 on the same positions. First of all, a distinct deuterium line at $656.1\,{\mathrm{nm}}$ is detected in the exposed tile (left), while in the unexposed tile (right) only the hydrogen line at $656.28\,{\mathrm{nm}}$ can be observed. The presence of the hydrogen line in both tiles is most likely attributed to adsorbed moisture on the surface and hydrogen remaining in the bulk. Moreover, it is important to point out that after the first laser pulse no Balmer-$\alpha$ line can be detected. Only the atomic tungsten line at $656.32\,{\mathrm{nm}}$ can be observed. Applying a pseudo-Voigt fit of the superposition of $\text{H}_\alpha , \text{D}_\alpha$ and the W-I line allows us to compare the broadening effects on the heavy and regular hydrogen atoms. Here, we observe FWHM of $\Delta \lambda _{H}=231\,{\mathrm{pm}}$ and $\Delta \lambda _{D}=140\,{\mathrm{pm}}$ respectively. This discrepancy can be explained by different reduced masses $\mu$ of the collision partners influencing the Stark effect and the dependency of the Doppler width on the atomic mass as $\sim \sqrt{m^{-1}}$. It is known that combination of collision and temperature effects are responsible for the line broadening^26^.

Observing the corresponding crater for this LIBS measurement provides information on the possible ablation rate that can be applied to this setup. Figure 5 shows a typical crater produced by five single laser pulses in argon environment on the W-tile. We find that the ablation depth per pulse (depth resolution) that allows us to observe signals from low-level hydrogen and deuterium concentration is $600\,{\mathrm{nm}}$. In this configuration the laser beam diameter $D_0$ is given as $20\,\upmu \mathrm{m}$. Integration over the whole crater brings the total ablated volume of $(282\pm 25)\,\upmu \mathrm{m}^3$ for five consecutive laser pulses.

The combination of all presented data on the temporal plasma emission dynamics and the possibility to separate the hydrogen isotopes in the first laser pulse gives us the opportunity to estimate the deuterium impact to the ablation yield. Here, a quantitative investigation on the deposition is possible and given for the tungsten tiles. The measured temperature can be used to calculate the deuterium and hydrogen concentration by plotting the measured intensity of the hydrogen Balmer-$\alpha$ line to the Boltzmann-plot from equation (3). Here, it is assumed that the tungsten and hydrogen subsystems exhibit the same temperature calculated by the Boltzmann-plot method. The used spectral characteristics of the $\text{H}_\alpha$ line are presented in Table 1. Note that the measured intensity needs to be adapted to the sensor sensitivity calibrated by a halogen lamp of defined emission characteristics. The estimations for CF-LIBS mentioned in the methods section are applied to the calculation. In addition, the composition of the observed plasma in a LTE (delays longer than $400\,{\mathrm{ns}}$) is assumed to be purely tungsten, hydrogen and deuterium atoms. Further impurities on the surface and in the bulk, and the argon atmosphere are ignored. From the intercept of the Boltzmann-plot the concentration of tungsten $C_W$ and both hydrogen isotopes $\text{H}$ and $\text{D}$ combined $C_{H \& D}$ can be estimated. The evaluated fraction is given as

1 $$\begin{aligned} \%(\text{H}+\text{D})=\frac{C_{H \& D}}{C_{H \& D}+C_W}\cdot 100\,\%. \end{aligned}$$

From the high spectral resolution measurement in Fig. 4 and the pseudo-Voigt fits, a ratio of the integrated peak-areas is calculated as $\text{D}_{\alpha }/\text{H}_{\alpha }\approx 0.08$. This relation is transferred to the total number ratio of deuterium and hydrogen atoms $N_D/N_H$ and can then be used to estimate the total deuterium concentration. With a total ablated volume of $(57\pm 5)\,\upmu \mathrm{m}^3$ per laser pulse, as estimated from the findings in Fig. 5, the total number of ablated tungsten atoms is calculated as $N_W=(3.6\pm 0.3)\times 10^{12}$. Taking into account the molar volume of tungsten as $M_{V,W}=9.47\times 10^{-6}\,\mathrm{m}^{3}{\mathrm{mol}}^{-1}$ and applying Eq. (1), the found concentration values and the total number ratio, the number of hydrogen and deuterium atoms were calculated as $N_H=(7.8\pm 3.9)\times 10^{11}$ and $N_D=(6.2\pm 2.8)\times 10^{10}$ respectively, as average values determined by the detected spectra with delay $>400\,{\mathrm{ns}}$. Here, the uncertainties are just statistical variations and have to be extended by the mentioned approximations and deviations of the ablated volume. This includes that the value is probably more an upper limit due to the overestimated Balmer-$\alpha$ intensity that is influenced by a $\text{W}$-I line. Considering this, the value holds up to a comparison to the TDS data. The total number of deuterium atoms detected in the whole sample is estimated as $(3.8\pm 0.8)\times 10^{16}$. Here, an accuracy of around $21\%$ is calculated. From this we expect up to $(1.2\pm 0.2)\times 10^{11}$ atoms on the laser irradiated spot in the LIBS experiment, which is a factor two larger than what we calculated by the CF-LIBS approach. This deviation might result from the uncertainty of the two methods, as it can be expected from studies on PSI-2^27^ that deuterium is only stored in depths of around $100\,{\mathrm{nm}}$. Also note that the deuterium distribution along one dimension of the tiles surface is not homogeneous due to the plasma gradient given in the exposure process. This can result in an over or underestimation of the expected deuterium number depending on the position on the tile. The presented measurement is executed close to the center of the tile and along the axis where we do not expect significant changes in the deposition. In conclusion, the presented CF-LIBS method can be used to determine the deuterium impact in the used W tiles as around $(1.7\pm 0.5)\,\text{at}\%$ in the first $600\,{\mathrm{nm}}$ behind the surface with a high lateral resolution of $\sim 20\,\upmu \mathrm{m}$, according to the crater diameter.

## Conclusion

We demonstrated the use of femtosecond UV LIBS as a prospect high depth resolution diagnostic technique to analyze the hydrogen isotope impurities in metallic samples that are used as PFCs in confinement fusion experiments. With an ablation rate of $600\,{\mathrm{nm}}$ per pulse, deuterium and hydrogen can be detected with this method. Here the limitation of femtosecond LIBS can be observed compared to studies with picosecond lasers that provide a higher pulse energy. In particular, the studies by Oelmann et al. (2021)^28^ exhibit a depth resolution of $30\,{\mathrm{nm}}$ in a double-pulse configuration. With an enormously shorter heat entry to the sample the heat affection by the laser is smaller and desorption of light particles from higher depths is less likely in relation to ns-LIBS experiments. This will influence quantitative approaches like Xing et al.^29^, because the ablated volume can not be consulted to calculate the deposited deuterium fraction. The applied CF-LIBS method here is an interesting quantitative approach to estimate the total deuterium content in the investigated tungsten tiles and is even more significant due to the use of the femtosecond laser to provide a reasonable depth and high lateral resolution. The estimated deuterium content of approximately $1.7\,\text{at}\%$ using this method is close to the expected quantity in the tungsten tiles that were exposed to deuterium plasma. Moreover, similar concentrations of hydrogen retention was found like in the comparable study by Pardede et al.^30^. Further studies on this approach with tiles of varying deuterium content would be the next logical step to develop this method and to determine the limit of detection. In addition, interesting approaches to enhance the detected plasma emission will be beneficial to improve the signal-to-noise ratio^31^. In conclusion, this work provides insights into the use of all-optical laser plasma techniques for future in-situ analysis of plasma-facing components in fusion applications.

## Methods

In the following, the used experimental setup, evaluation methods, and sample preparation techniques are described. For more details on the determination of ablation threshold fluence of tungsten, and the results from the fitting function, refer to the provided supplemental information.

### Experimental setup

**Figure 6:**
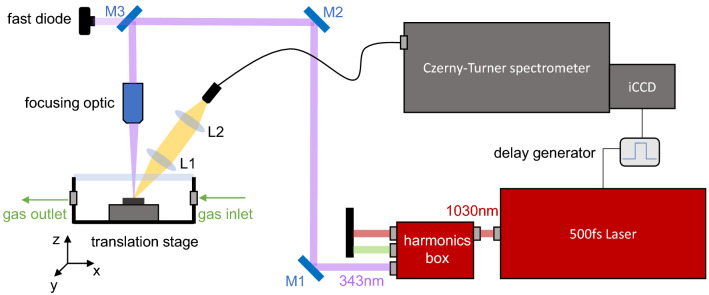
Experimental Setup of femtosecond UV LIBS experiment including the laser system, harmonic generation box, a set of mirrors (M1-M3), the focusing optic, a translation stage with the sample inside of a chamber with a gas in- and outlet, and the collection system with two lenses (L1 and L2) and a Czerny-Turner spectrometer with iCCD camera.

The basic setup of the LIBS experiment is shown in Fig. 6. It consists of a $\lambda =1030\,{\mathrm{nm}}$, $500\,\mathrm{fs}$ laser in single pulse mode and a setup for second and third harmonic generation. In this set of experiments, the third harmonic of the fundamental laser frequency was used at $343\,{\mathrm{nm}}$. The generated pulses with an output pulse energy of up to $100\,\upmu \mathrm{J}$ are focused by a 3x objective lens (working distance $50\,{\mathrm{mm}}$) to the target placed in an experimental cell ($10\,{\mathrm{cm}}\times 10\,{\mathrm{cm}}\times 5\,{\mathrm{cm}}$) that can be filled with different gas compositions from an external inlet. Here an argon gas flow of $2\,{\mathrm {l/min}}$ is chosen to change the environmental conditions. The whole cell is placed on motorized stages to control the distance between the focusing lens and the target and to irradiate different positions on the surface. A collection system consists of two plano-convex fused silica lenses ($f_1=50\,{\mathrm{mm}}$ and $f_2=100\,{\mathrm{mm}}$) and an optical fiber that is coupled to the setup to collect the plasma radiation and image it to the entrance slit of a Czerny-Turner spectrometer. The plasma emission signal is detected by an iCCD camera.

In this work, we studied the time resolved UV femtosecond LIBS emission from tungsten tiles that were exposed to a deuterium plasma in a linear plasma device, and followed by the detection and quantification of the hydrogen isotopes in these samples.

### Preparation of W-tiles

The material under investigation is pure polished tungsten (W, $Z=74$) with a surface roughness of $S_a=60\,{\mathrm{nm}}$. The tiles ($9.9\,{\mathrm{mm}}\times 9.9\,{\mathrm{mm}}\times 5.1\,{\mathrm{mm}}$) are baked out under $1000\,^\circ \mathrm{C}$ for three hours and exposed to deuterium in the linear plasma device PSI-2 at Forschungszentrum Jülich. The tiles are arranged in a circle on a molybdenum mask while the ring shaped plasma interacts with it. Plasma parameters are detected by a Langmuir probe frequently during the four hour process. The maximum deuterium flux is measured as $2.9\times 10^{21}\,\mathrm{m}^{-2}\mathrm{s}^{-1}$ with a total fluence of $3\times 10^{25}\,\mathrm{m}^{-2}$ onto the tile surface heated to a temperature of $230\,^\circ \mathrm{C}$. The plasma parameters in the PSI-2 to mimic the fusion plasma are described by Kreter et al.^27^. From this overview, a deuterium concentration of up to $2\,{\mathrm {at\%}}$ in the first $1\,\upmu \mathrm{m}$ behind the surface is reasonable with the used settings. According to a Thermal Desorption Spectroscopy (TDS) measurement applied ex-situ after the exposure, the total number of detected deuterium atoms deposited per area in the bulk are given as $N_D=(3.9\pm 0.8)\times 10^{20}\,\mathrm{m}^{-2}$, and $N_H=(4.1\pm 1.1)\times 10^{21}\,\mathrm{m}^{-2}$ hydrogen atoms. The ratio of $N_D$ to $N_H$ is with 0.095 close to the calculated value from the high resolution LIBS experiment.

### Evaluation of the results

In this section we give an overview of the used methods to evaluate the measured spectra and how to gain more information from the samples under investigation. Calculations of plasma parameters such as temperature and electron number density are necessary to resolve a spectrum from hydrogen and deuterium impurities in the used metal tiles. Cristoforetti et al.^25^ showed that the expanding, transient plasma created by the laser pulse and interacting with the ambient gas can be in a LTE under certain circumstances. This state is a necessary condition to make predictions on the plasma parameters. Following the conditions formulated there and the criterion given by McWhirter et al.^32^,

2 $$\begin{aligned} n_e>1.6\times 10^{12} \left( T[\mathrm{K}]\right) ^{1/2} \left( \Delta E_{mn}[{\mathrm{eV}}]\right) ^3, \end{aligned}$$

the tenability of this state is reviewed. Note that the McWhirter criterion alone is not sufficient in this context.

The acceptance of a LTE allows the application of the Saha equation and a Maxwellian velocity distribution on the electrons in the expanding plasma. Moreover, this implies that the temperature of electrons and all other particles in the plasma plume are equal ($T_e=T_h$) and we want to assumed that it is optically thin. In this case we can apply the linear form of the Boltzmann-plot method as

3 $$\begin{aligned} \ln {\left( \frac{\lambda _{ki}I_{ki}}{hcA_{ki}g_k} \right) }=-\frac{E_k}{k_BT}+\ln {\left( \frac{FC_s}{U_s(T)}\right) }. \end{aligned}$$

In this equation the indices *k* and *i* represent the upper and lower state of the excited species respectively, the natural constants are given as *h* for Planck, $k_B$ for Boltzmann and *c* for the vacuum light velocity. Additional parameters for the transition wavelength $\lambda _{ki}$ like transition probability $A_{ki}$, statistical weight $g_k$, and upper energy level $E_k$ can be extracted from common literature (e.g. NIST library^18^). $U_s(T)$ is the temperature dependent partition function that can be calculated according to the statistical weight and energy levels given for each material from the same library. Note that the experimental factor *F* is the same for all analyzed materials in the same plasma as it depends on the collection system and plasma size. The linear character ($y=mx+q_s$) of this form allows the extraction of the temperature value from the slope $m=-(k_BT)^{-1}$ by plotting the data on a logarithmic axis. In the intercept we can find the information on the species concentration $C_s$ to the extend of the experimental factor *F*. This fact can be used in a Calibration Free LIBS method (CF-LIBS) to estimate the amount of ablated deuterium in the detected plasma matrix. The application of this is discussed in detail in the experimental result section. For now we assume that the ablated material contribution can be mapped by the spectral emission distribution. Specifically, we work under the assumption of stoichiometric ablation^33,34^, that the observed plasmas (in a reasonable time frame) are optically thin, and that the used spectral lines for the Boltzmann-plot method are not self-absorbing.

The evaluation of the electron number density is made by analysis of the spectral line broadening of the hydrogen Balmer-$\alpha$ emission. The spectral line shape is determined by the convolution of different effects, where natural line broadening and van-der-Waals broadening are neglected and the instrumental broadening is determined for every measurement. The two dominant effects are the Doppler broadening, which is a temperature effect, and the Stark broadening induced by collisions of the charged particles, especially electrons, with the other parts present in the plasma. Due to the assumed Maxwell-Boltzmann velocity distribution the spectral lines are influenced by the plasma temperature and a Gaussian shape of width

4 $$\begin{aligned} w_{Doppler}=\frac{\lambda _0}{c}\sqrt{2\ln {2}\frac{k_BT}{m_s}}, \end{aligned}$$

with spectral line wavelength $\lambda _0$ and species mass $m_s$, can be observed. This width is given as the full width at half maximum (FWHM) divided by the factor $2\sqrt{2\ln {2}}$. The Stark broadening follows a Lorentz profile and the electron number density $n_e$ can be estimated by the half width $w_{Stark}$, also called Stark width, and the Stark broadening parameter $w_0$ that can be found in literature. Note that $w_0$ also depends on the plasma temperature. It has been shown in^35,36^ that it is possible to neglect the temperature dependence by determining the Half Width at Half Area (HWHA) of the deconvoluted Lorentz part of the Balmer-$\alpha$ emission line and calculate $n_e$ by

5 $$\begin{aligned} \text{HWHA}[{\mathrm{nm}}]=0.549\cdot \left( \frac{n_e[{\mathrm{cm}}^{-3}]}{10^{17}}\right) ^{0.67965}. \end{aligned}$$

Taking into account the convolution of the Gaussian and the Lorentz shape, a pseudo-Voigt fit as described in^37^ is used in this contribution to extract the plasma parameter from the line shape characteristic. We found that this fitting method was significantly more stable in the data processing compared to a real Voigt fit, also with data sets of lower signal-to-noise ratio. More information on the fitting function can be found in the SI.

## Supplementary Information

Supplementary Information.

## Data Availability

The experimental datasets generated during the current study are available from the corresponding author on reasonable request.
