# Supplementary material for: Hydrogen isotope analysis in W-tiles using fs-LIBS
Source: Sci Rep. 2023 Feb 9;13:2285. doi: 10.1038/s41598-023-29138-2 (PMC9911398; doi:10.1038/s41598-023-29138-2)
Supplement: Supplementary file 1 — Supplementary Information. [file 41598_2023_29138_MOESM1_ESM.pdf]

Supplemental Information

Laser Ablation of W

In addition to the optical emission experiments on the expanding plasma, we want to give a classification of the used laser fluence. For this we present an ablation experiment to estimate the threshold fluence of the used laser on polished tungsten samples. Moreover, a definition of the used laser fluence is given.
Analogous to Ref. [1] the used laser peak fluence of the almost top-hat beam profile can be described by the pulse energy E and beam diameter D_0 at $1/e^2$ of the maximum intensity as

$$F = \frac{4E}{\pi D_0^2}. \tag{1}$$

The diameter is estimated by observing the produced crater morphology. Moreover, following the theory of Chichkov et al. (1996) [2] the threshold fluence F_{th} can be estimated by applying the formula

$$L = \alpha^{-1} \ln\left(\frac{F}{F_{\mathrm{th}}}\right) \tag{2}$$

as a fit to the ablation rate L (depth per pulse) depending on the varying laser fluence. Here α^{-1} is a numerical fitting parameter that can be interpreted as the optical penetration depth. Similar to the analysis of Lickschat et al. (2020) [3] the threshold fluence of ultra-short laser pulses can be estimated as

$$F_{th} = \frac{H_v \cdot \rho}{(1 - R(\lambda)) \cdot M \cdot \alpha(\lambda)}. \tag{3}$$

The necessary material parameters for the bulk tungsten (W) are given as: Evaporation enthalpy $H_v = 774\,\mathrm{kJ/mol}$, density $\rho = 19.25\,\mathrm{g/cm^3}$, molar mass $M = 183.84\,\mathrm{g/mol}$ and wavelength dependent values of reflectivity $R(343\,\mathrm{nm}) = 0.34$ and optical penetration depth $\alpha(343\,\mathrm{nm}) = 9.26 \times 10^5\,\mathrm{cm^{-1}}$ [4]. The resulting theoretical threshold fluence is calculated as $F_{th} = 0.13\,\mathrm{J/cm^2}$.
The laser induced craters by multiple laser irradiations on the same position are observed by the white light interferometer *Zygo NewView 6000 3D* that is also used to determine the surface roughness of the used tiles. The surface roughness of the tiles under investigation is given as $S_a = \frac{1}{N}\sum_{k=1}^{N} |z_k - u|$ with mean height u over N positions. This results in $S_a = 60\,\mathrm{nm}$ for the tungsten tiles used in the described experiments. In Fig. S1 the fluence dependent ablation rate is given for the low laser fluence regime on a bulk tungsten sample. Here craters are formed by ten consecutive laser pulses on the same position and ablation rate is estimated by

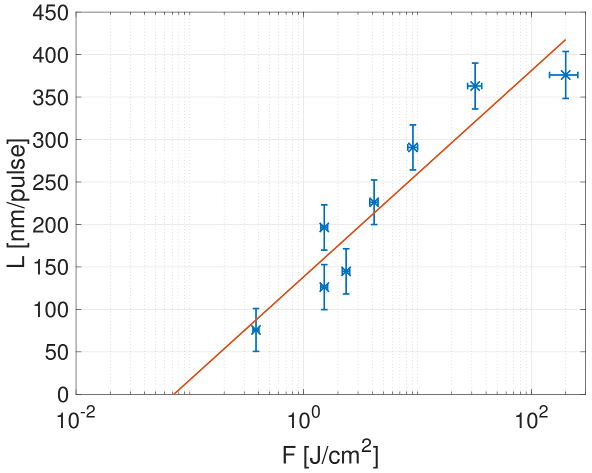

Fig. S1: Ablation on tungsten (W) using the single pulsed Amplitude laser ($\lambda = 343\,\mathrm{nm}, 500\,\mathrm{fs}$). Here the logarithmic fitting function of ablation rate L with respect to laser fluence F is shown in orange.

crater depth divided by the number of pulses. The output power is reduced to $30\,\mu\mathrm{J}$ by a half wave plate and a beamsplitter. Moreover the fluence is controlled by variation of objective lens and surface distance that changes the beam diameter. The presented errorbars on the measurement (blue crosses) result from statistical uncertainties measuring nine craters for each fluence and systematical uncertainties by the knowledge of pulse energy by approximately $\pm 5\,\%$, beam diameter by $\pm 1\,\mu\mathrm{m}$ and crater depth by $\pm 50\,\mathrm{nm}$. Applying equation (2) as a fit function using F_{th} and α as fitting parameters, the resulting threshold fluence is given as $F_{th} = (0.07 \pm 0.06)\,\mathrm{J/cm^2}$. This value is consistent with the overestimated calculated value of $0.13\,\mathrm{J/cm^2}$ from equation (3). Note that the surface roughness might have an impact on the used reflectivity value in this estimation.

Fitting Function

Regarding the determination of plasma parameters and the detection of the isotopic shift, the used fitting process is of major interest and is presented here in more detail. First, the de-convolution of the pseudo-Voigt fitting function to the H_α line detected with the spectrometer of lower resolution is shown. Second, the fitting to the isotopic shifted lines detected with the high resolution spectrometer is discussed.
In Fig. S2 the de-convoluted Gauss and Lorentz part included in the pseudo-Voigt fit to the H_α line detected with a delay of $200\,\mathrm{ns}$ is shown as an example. Here

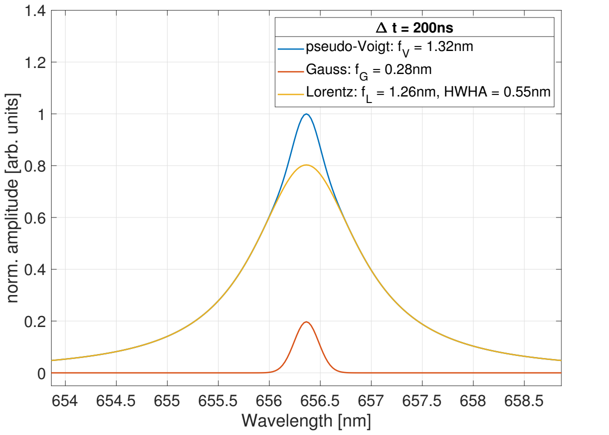

Fig. S2: De-convolution of the pseudo-Voigt fit in Gauss and Lorentz part to the H_α line detected with a delay of 200 ns. FWHM of the de-convoluted curves are given as f_V, f_G, and f_L.

f_V, f_G, and f_L are assigned as the FWHM of the three curves and the Half Width at Half Area (HWHA) or the Lorentz part is given here. In the paper, the plasma temperature is determined by the Gaussian width, because it refers to the Doppler Broadening, and the number density is calculated by the HWHA of the Lorentz part.
In the presented CF-LIBS approach the exact spectral line shape of the isotopic shift in Fig. S3 is of main interest to identify the correct hydrogen and deuterium ratio and corresponding plasma parameters. Here we want to give more insights to the used pseudo-Voigt fitting function that we used. As mentioned in the methods part, the fitting follows the routine in Ref. [5]. Here the pseudo-Voigt function is given as a linear superposition of a Lorentz ($L(x, f_L)$) and a Gaussian ($G(x, f_G)$) function as

$$V(f_L, f_G) = \eta \cdot L(x, f_L) + (1 - \eta) \cdot G(x, f_G). \tag{4}$$

Here η is a weighting parameter given as

$$\eta = 1.36603 \cdot \frac{f_L}{f} - 0.47719 \cdot \left(\frac{f_L}{f}\right)^2 + 0.11116 \cdot \left(\frac{f_L}{f}\right)^3, \tag{5}$$

where f is given as

$$f = (f_G^5 + 2.69269 \cdot f_G^4 \cdot f_L + 2.42843 \cdot f_G^3 \cdot f_L^2 + ... \\ 4.47163 \cdot f_G^2 \cdot f_L^3 + 0.07842 \cdot f_G \cdot f_L^4 + f_L^5)^{1/5}. \tag{6}$$

Note that f_G and f_L are the FWHM of the Gauss and Lorentz part that are given as

$$G(x, f_G) = \frac{2\sqrt{\ln 2}}{f_G\sqrt{\pi}} \cdot \exp\left(-4\ln 2\frac{x^2}{f_G^2}\right) \tag{7}$$

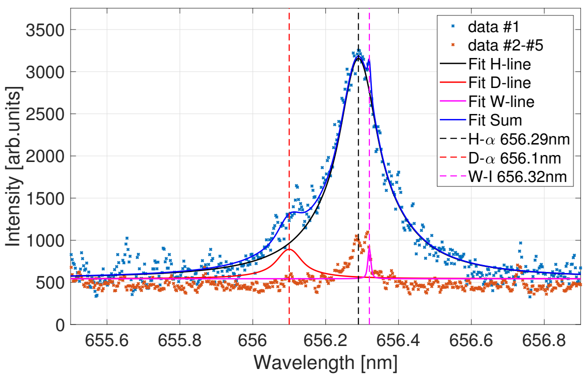

Fig. S3: Pseudo-Voigt fit on Hydrogen and Deuterium spectral line detected in tungsten (see Fig. 5 in the paper).

and

$$L(x, f_L) = \frac{f_L}{2 \cdot \pi \cdot (x^2 + (f_L/2)^2)} \tag{8}$$

respectively. For the fit-function of the two isotopes we used

$$F(\lambda) = b + h \cdot V(f_{G,H}, f_{L,H}, \lambda - \lambda_H) + ... \\ d \cdot V(f_{G,D}, f_{L,D}, \lambda - \lambda_D) + ... \\ w \cdot V(f_{G,W}, f_{L,W}, \lambda - \lambda_W) \tag{9}$$

with $f_{G,H}$, $f_{G,D}$, $f_{L,H}$, $f_{L,D}$, h, d, and the offset b as fitting parameters with fixed central wavelengths $\lambda_H =$ 656.28 nm, $\lambda_D =$ 656.1 nm, and neutral tungsten line with $f_{G,W}$, $f_{L,W}$ of amplitude w at $\lambda_W =$ 656.32 nm. Here V is a normalized function. Note that the ratio $f_{G,H}/f_{G,D} = \sqrt{2}$ is given due to the mass dependent doppler broadening. The calculated fitting parameters are given with a 95 % confidence bound as

$$\begin{aligned} f_{G,H} &= 0.07862\,\text{nm} \\ f_{G,D} &= 0.0556\,\text{nm} \\ f_{G,W} &= 0.01\,\text{nm} \\ f_{L,H} &= 0.2041\,\text{nm} \\ f_{L,D} &= 0.1183\,\text{nm} \\ f_{L,W} &= 0.01\,\text{nm} \\ h &= 600 \\ d &= 349.38 \\ w &= 358.6 \\ b &= 543.10 \end{aligned} \tag{10}$$

The presented FWHM in the paper ($\Delta\lambda_H = 231\,\mathrm{pm}$ and $\Delta\lambda_D = 140\,\mathrm{pm}$) correspond to the full width of the pseudo-Voigt calculated as

$$f_V = \frac{f_L}{2} + \sqrt{\frac{f_L^2}{4} + f_G^2}. \tag{11}$$

From this fit the plasma parameters can be extracted as described and the hydrogen-deuterium composition is calculated by the ratio of the integrals over the two pseudo-Voigt functions.

References

1. Mittelmann, S., Oelmann, J., Brezinsek, S., Wu, D., Ding, H., and Pretzler, G. (2020). Laser-induced ablation of tantalum in a wide range of pulse durations. Applied Physics A, 126(9), 1-7.
2. Chichkov, B. N., Momma, C., Nolte, S., Von Alvensleben, F., and Tünnermann, A. (1996). Femtosecond, picosecond and nanosecond laser ablation of solids. Applied physics A, 63(2), 109-115.
3. Lickschat, P., Metzner, D., and Weißmantel, S. (2020). Fundamental investigations of ultrashort pulsed laser ablation on stainless steel and cemented tungsten carbide. The International Journal of Advanced Manufacturing Technology, 109(3), 1167-1175.
4. Werner, W. S., Glantschnig, K., and Ambrosch-Draxl, C. (2009). Optical constants and inelastic electron-scattering data for 17 elemental metals. Journal of Physical and Chemical Reference Data, 38(4), 1013-1092.
5. Ida, T., Ando, M., and Toraya, H. (2000). Extended pseudo-Voigt function for approximating the Voigt profile. Journal of Applied Crystallography, 33(6), 1311-1316.
